# Suicide Behavior Before and After the Start with Antidepressants: A High Persistent Risk in the First Month of Treatment Among the Young

**DOI:** 10.1093/ijnp/pyv081

**Published:** 2015-07-18

**Authors:** Fabian Termorshuizen, Saskia JM Palmen, Eibert R Heerdink

**Affiliations:** Division of Pharmacoepidemiology & Clinical Pharmacology, Utrecht Institute of Pharmaceutical Sciences, The Netherlands (Drs Termorshuizen and Heerdink); Julius Center for Health Sciences and Primary Care, University Medical Center Utrecht, The Netherlands (Dr Termorshuizen); Brain Center Rudolf Magnus, Department of Psychiatry, University Medical Center Utrecht, The Netherlands (Dr Palmen); Altrecht Institute for Mental Health Care, Utrecht, The Netherlands (Dr Heerdink); Department of Clinical Pharmacy, University Medical Center Utrecht, The Netherlands (Dr Heerdink).

**Keywords:** age, antidepressants, pharmaco-epidemiology, suicide, suicide attempts

## Abstract

**Background:**

A causal relationship between antidepressants (ADs) and a high risk of suicidal behavior at a young age has been suggested. We analyzed the rates of suicide attempts during treatment with AD in comparison with the rates before treatment initiation for different ages.

**Methods:**

Claims of insurance company Achmea were linked to the population registry of Statistics Netherlands. Episodes of AD use were defined for those with their first registered prescription in 2006–2011 (n = 66,196). Rates were analyzed in a Poisson model. Correlates of attempts in the first month of AD use were assessed in a logistic model.

**Results:**

Among those aged <25 years, a high rate of suicide attempts during the month before the start of ADs was found (376.3/10 000 person yrs). A non-significant increase in the first month (*p* = 0.212) was found and a non-significant trend to lower values was determined thereafter (*p* = 0.3050). Among those ≧25 years, a clear decrease to lower rates immediately after the start was observed (*p* < 0.025). The highest rates of suicide were found among those >40 years during the first month. Female gender was, but treatment characteristics were not, associated with early attempts at a young age.

**Conclusions:**

Among young AD users, a high pre-treatment risk of suicide attempts was present and persisted during the early phases after the start. This contrasted with the clear decrease in risk among those aged ≧25 years, suggesting lower effectiveness of ADs to prevent suicidal behavior at young ages. Caution should be exercised to infer a causal relationship or to use data on attempts to predict risk of suicide during AD use.

## Introduction

The paradoxical association between use of antidepressants (ADs), especially the selective serotonin reuptake inhibitors (SSRIs), and an increased risk of suicidal behavior, in particular among the young, is still a matter of debate. The Food and Drug Administration’s reanalyses of clinical trials suggest adverse effects on suicide ideation and attempts at young ages (Hammad et al., 2006; Stone et al., 2009) and ecological studies report a reverse association with both attempts and suicide as cause of death (Gibbons et al., 2007a; Gusmao et al., 2013; Lu et al., 2014), leaving psychiatrists and other health care providers in a cloud of uncertainty regarding the safety of ADs.

A number of observational studies have shown that ADs may be associated with an increased risk of suicide attempts among the young, but results are sensitive to confounding by indication (Martinez et al., 2005; Olfson et al., 2006; Tiihonen et al., 2006; Miller et al., 2014). That is, the higher risk of suicide behavior may be explained by the severity of the underlying disorder and not necessarily by an effect of the treatment. If a causal relationship between ADs and suicide behavior is present, this is probably restricted to the early stages following the initiation (Morrison and Schwartz, 2014). In a study by Jick et al. (2004) among patients with a broad age range in which data from the UK General Practice Research Database were used, the risk of attempts was four times higher during the 9 days following the start of ADs compared to later stages. And in a study by Valuck et al. (2004) among adolescents aged 12–18 years using a US database with health insurance claims, the hazard of suicide attempts decreased significantly with a longer treatment duration (>180 days). Still, these findings may reflect the severity of the mental disorder in the early phases, when ADs have not fully exerted their beneficial effects yet. Therefore, it is important to include the risk of suicide attempts before the start of ADs in the analyses. Few studies have done this. In the population-based record-linkage study of Simon et al. (2006) using US pharmacy records and hospital admission data, the highest risk of suicide attempts was found in the month preceding the initiation of AD use and a consistent decline was noted thereafter. At ages <18 years, the risk of attempts was high compared with older age groups, but this was found already before AD initiation and a consistent decline thereafter was established in all age groups.

We compared the rate of suicide attempts following the start of ADs with the rate before the start among incident users. We assessed whether age might modify this comparison, and what patient and treatment characteristics were associated with the risk of suicide attempts in the first month after the start of ADs.

## Methods

### Databases

In this retrospective cohort study, two data sources were used. The first source of data was the Achmea Health Insurance Database (AHD). Achmea is one of the largest health insurance companies in the Netherlands, with policyholders spread over the country but with the center of the Netherlands as one of the dominant regions. Although it is a private company, insurance for the provision of medical care is compulsory for all inhabitants in the Netherlands, and thus the data from the Achmea Health Database may be regarded as reasonably representative for the health care utilization of the Dutch population. In the AHD, all reimbursements for the provision of medical care to the insured patients of Achmea are recorded. This includes drug prescriptions delivered by pharmacists and so-called Diagnostic Treatment Protocols (in Dutch, Diagnose Behandel Combinatie; DBC). A DBC is an insurance claim containing codes for diagnosis and treatment by a medical specialist. The second data source was the population register and the linked death register of Statistics Netherlands (in Dutch, Centraal Bureau voor de Statistiek; CBS). The CBS is responsible for collecting and processing individual and population health care data in the Netherlands. Physicians in the Netherlands are obliged to report the cause of death to the civil registry of the town where the person died. This is forwarded to the CBS, where the death report is coded according to the ICD-10 (International Statistical Classification of Diseases and Related Health Problems 10th revision). Dutch privacy law allows use of these data sources for scientific research under strict conditions in relation to anonymity and storage, in which case informed consent is not needed.

### Patients and Data Extraction

For the present analysis, we used data from a large database with prescriptions of antidepressants. For 232 561 patients with at least one AD prescription in 2001–2011 following a registered period of at least 1 year without AD use, episodes of AD use and intermittent episodes of no use could be defined. The duration of these episodes was assessed by dividing the cumulative number of defined daily dosages by the prescribed daily dosage (PDD) of each prescription. Two consecutive prescriptions were regarded as belonging to the same ongoing treatment episode if the time gap between them was ≤14 days. Both fatal and non-fatal suicide events were assigned to the episode of AD use or to the episode of no use during which they occurred. For suicide, the registered codes X60-84 (intentional self-harm) and Y10-34 (event of undetermined intent) of the ICD-10 were used. For suicide attempts, claims with a DBC code for specialism 0329 (psychiatric consultation in hospital) in combination with a code for care demand 01 (suicide attempt) or for specialism 0313 (internal medicine) in combination with a code for a diagnosis 042 (auto-)intoxication were included. For the present study, we restricted the analysis to those patients with their first AD prescription in the period from July 2006 until June 2011 (n = 66 196). This time period was chosen because the system for registration of DBC’s became operational from January 2006 onwards, meaning 6 months of observational time prior to the start of AD treatment and a maximum of 6 months thereafter were available for all selected patients. For the selected patients, suicide attempts during the 6 months preceding the first registered AD prescription were extracted from the AHD and added to the database. Each of these attempts was assigned to one of the six predefined 1-month episodes during which the attempt occurred. The earlier defined episodes of use and (intermittent) episodes of no use during the first half year following the first AD prescription were also split up into six 1-month episodes. Episodes of no use started after 14 days following the estimated end date of the last dispensed prescription (see above) and ended at the date of a new AD prescription. The large database with data from 2001 onwards will be used in a sequel of the present study to investigate the relationship by age category between episodes of antidepressant use and suicide as the cause of death.

### Analysis

Crude rates (n of events/10 000 person years) were calculated by dividing the number of registered suicide attempts by follow-up time. This was done separately for each 1-month episode during the 6 months before and the 6 months after the start with ADs, and separately for different age categories. Furthermore, for the 1-month episodes after the start, a distinction was made between observation time during use of ADs and observation time during (intermittent) episodes without ADs. In a Poisson regression analysis, the ratios of the rates for the 1-month episodes after the start during actual use of ADs versus the rate during the month immediately prior to the start (the reference rate) were tested. Terms for age x month interaction were included to estimate different sets of incidence rate ratios (IRR) for different age categories. Possible dependence of the rates within the same patient was taken into account by inclusion of a random intercept. Analyses were adjusted for age, gender, and ethnic origin (Dutch, other Western, non-Western).

In a second step, those with a suicide attempt during the first month after the start (n = 74) were compared to those without (n = 66 011). This was restricted to those without a suicide attempt during the month prior to the start (n = 66 085). We assumed that if a causal relationship between use of ADs and suicidal behavior is present, suicide attempts causally related to ADs will be found mainly among these early events. Besides age, these early attempts may be specifically associated with certain patient and/or treatment characteristics, which may shed light on the nature of a possible association. In a multivariable logistic regression model, potential correlates of the suicide attempts shortly after the start were taken into account. Age, gender, class, type, dosage (PDD) of the prescribed AD, and type of prescriber (general practitioner [GP] vs. medical specialist or psychiatrist) were included as covariates. Whether the relationship between age and risk of suicidal behavior might be modified by the presence of one of these third factors was explored by inclusion of terms for age x third factor interactions: that is, when the high suicide risk at a young age is found only or mainly among users of fluoxetine, this may result in a statistically significant age x fluoxetine interaction.

Data management, record linking, description of the study cohort, and estimation of crude suicide attempt rates were performed using SPSS, version 14.0 (IBM Business Analytics). The random-intercept Poisson regression and logistic regression analyses were performed using STATA, version 12.0 (STATACorp, http://www.stata.com/company/).

## Results

### Description

The majority of the 66 196 patients were aged 25 years or older (91.5%), but still a substantial minority was aged <25 years (n = 5 636; Table 1). Details on the use of ADs during the first 6 months after the first registered prescription are given by age category in Table 2. Among those aged <25 years, use of ADs related mainly to SSRIs (>60%). At higher ages, use of TCAs and other ADs became more important. Among those aged <18 years, the prescriber at the start was often a medical specialist (50.8%). At higher ages, a general practitioner was more often the prescriber at the time of first AD prescription (eg, at >60 years the GP was the prescriber for 74.7%). In accordance with this finding, the percentage of persons with a record in specialist psychiatric treatment was higher among the young (>50%), and declined consistently thereafter.

**Table 1.** Description

	N (%)
Total	66 196 (100%)
Gender	
Male	26 147 (39.5%)
Female	40 049 (60.5%)
Age at the date of first antidepressant prescription (years)	
<18	997 (1.5%)
18–24	4 639 (7.0%)
25–39	16 604 (25.1%)
40–60	23 366 (35.3%)
>60	20 590 (31.1%)
Ethnic origin	
Dutch	39 847 (60.2%)
Other Western	5 289 (8.0%)
Non-Western	21 060 (31.8%)

**Table 2.** Characteristics of AD Treatment by Age Category

Age (years)	<18	18–24	25–39	40–60	>60
% of time during episodes of AD use associated with					
SSRI	67.7%	63.2%	56.9%	45.4%	37.7%
TCA	20.1%	14.1%	15.8%	27.9%	39.4%
Other ADs/ combination of ADs	12.2%	22.7%	27.3%	26.7%	22.9%
Type of prescriber (at the start)					
General Practitioner	49.1%	64.7%	69.4%	68.0%	74.7%
Medical Specialist/ Other	50.8%	35.4%	30.5%	32.0%	25.4%
% of time after the start associated with					
Episode of AD use	58.8%	54.5%	57.5%	56.3%	53.9%
(Intermittent) episode of no AD use	41.2%	45.5%	42.5%	43.7%	46.1%
Number of registered AD prescriptions					
1	22.2%	25.4%	21.3%	23.5%	28.4%
>1	77.8%	74.6%	78.7%	76.5%	71.6%
Presence of record in specialist psychiatric treatment					
No	30.8%	33.3%	39.6%	52.6%	74.8%
Yes, with mood disorder	19.9%	19.2%	21.2%	16.3%	5.7%
Yes, with other diagnosis	49.3%	47.4%	39.2%	31.0%	19.5%

AD, antidepressant; SSRI, selective serotonin reuptake inhibitors; TCA, tricyclic antidepressants.

### Crude Rates of Suicide Attempt Before and After the Start

**Figure 1.**
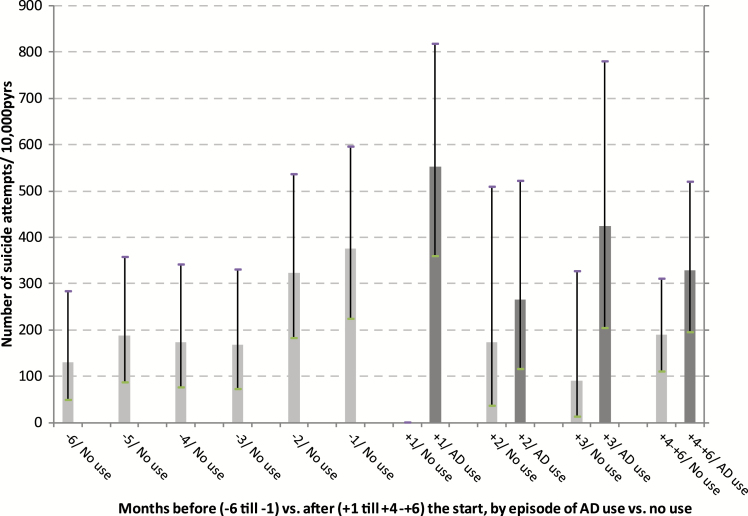
Number of suicide attempts per 10 000 person years of observation, by month before and after the start with ADs and by episodes of use and no use of ADs, for age group <25 years.

**Figure 2.**
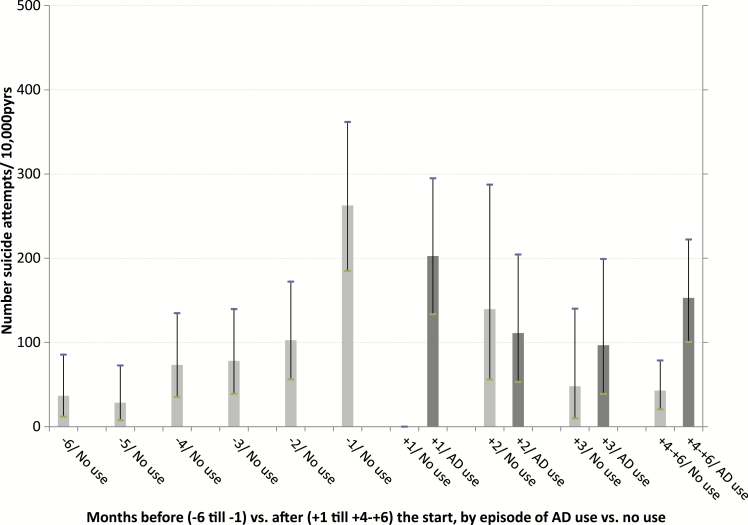
Number of suicide attempts per 10 000 person years of observation, by month before and after the start with ADs and by episodes of use and no use of ADs, for age group 25–40 years.

**Figure 3.**
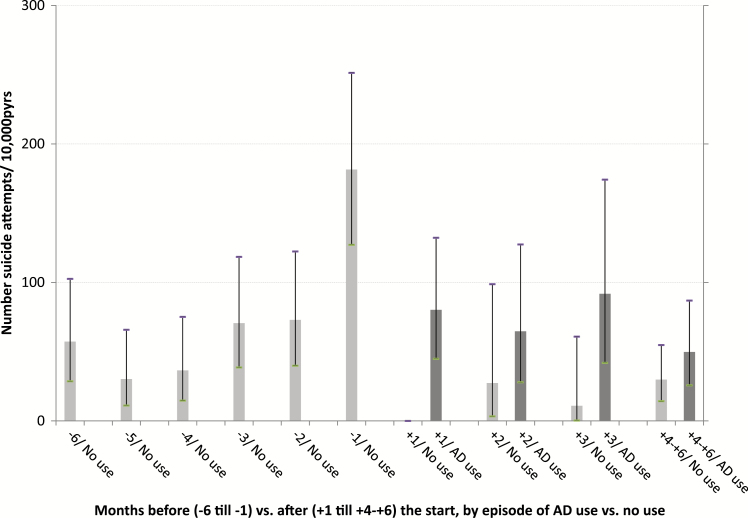
Number of suicide attempts per 10 000 person years of observation, by month before and after the start with ADs and by episodes of use and no use of ADs, for age group 40–60 years.

**Figure 4.**
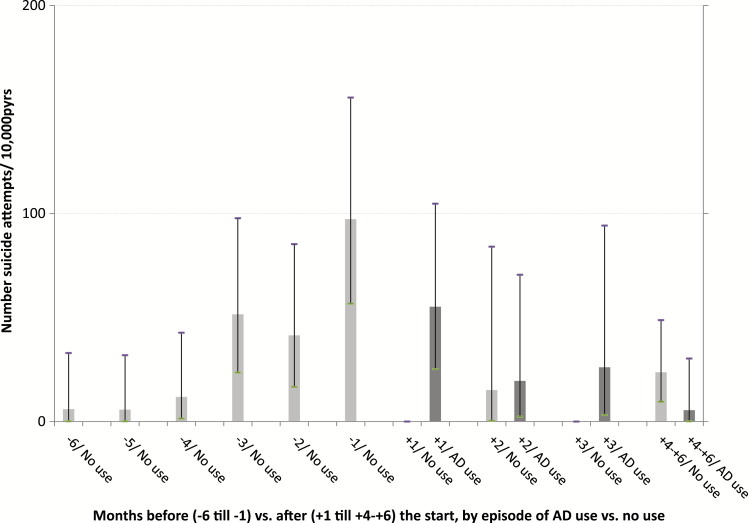
Number of suicide attempts per 10 000 person years of observation, by month before and after the start with ADs and by episodes of use and no use of ADs, for age group >60 years.

A number of 522 suicide attempts were registered during 65 468.4 person years of observation (pyrs). Following the first prescription, a distinction was made between observation time during episodes of AD use and observation time during (intermittent) episodes of no use. At each age category and during the 6 months after the first registered AD prescription, episodes of use were associated with suicide attempt rates that were higher compared to the rates during episodes of no use. Among those aged <18 and 18–24 years, high suicide attempt rates were found, both during the 6 months before and 6 months after the first AD prescription (Table 3, Figures 1–4).

**Table 3.** Number of Suicide Attempts by Age Category and Episode

Age category	Episode of AD use	Follow-up time (years)	Number of suicide attempts DBC 0329- 1101/ DBC 0313-042	Suicide attempt rate DBC 0329-1101 & DBC 0313-042 (N/10 000 person years) [95% Confidence Interval]
<18 years	6 mo. before first prescribed AD	499.5	8/ 5	260.2 [138.5–445.0]
	6 mo. after “: episodes of no use	202.4	0/ 0	-
	episodes of use	288.4	4/ 6	346.6 [166.2–637.4]
18–24 years	6 mo. before first prescribed AD	2 324.2	17/ 34	219.4 [163.3–288.5]
	6 mo. after “: episodes of no use	1 043.1	1/ 20	201.3 [124.6–307.7]
	episodes of use	1 249.9	10/ 41	408.0 [303.7–536.4]
25–39 years	6 mo. before first prescribed AD	8 319.0	22/ 59	97.3 [77.3–121.0]
	6 mo. after “: episodes of no use	3 494.6	1/ 19	57.2 [34.9–88.3]
	episodes of use	4 723.6	12/ 59	150.3 [117.3–189.5]
40–60 years	6 mo. before first prescribed AD	11 706.9	27/61	75.1 [60.2–92.6]
	6 mo. after “: episodes of no use	5 045.2	2/11	25.7 [13.7–44.0]
	episodes of use	6 498.9	14/30	67.7 [49.1–90.8]
>60 years	6 mo. before first prescribed AD	10 316.1	16/ 21	35.8 [25.2–49.4]
	6 mo. after “: episodes of no use	4 495.4	2/ 6	17.8 [7.6–35.0]
	episodes of use	5 260.5	1/ 13	26.6 [14.5–44.6]
total		65 468.4	137/ 385	79.7 [73.0–86.8]

AD, antidepressant; DBC, Diagnostic Treatment Protocols.

In Figure 1–4, the 6 months before and the 6 months after the first AD prescription are split up in separate 1-month periods. Crude rates of suicide attempts (n/10 000 pyrs) are given by month and by age category. Because of small numbers of events, similar rates, and overlapping confidence intervals (Table 3), the two youngest age categories and, for all age categories, the months 4–6 after the start, were lumped together. At each age category, a steady increase in the suicide attempt rate during the 6 months prior to the start was observed. At ages ≧25 years, the high suicide attempt rate in the month before the start (month -1) was followed by an immediate decline thereafter. At ages <25 year, high suicide attempt rates were found at month -1 (376.3/10 000 pyrs), without difference by gender (males, 376.0; females, 376.4). During the first month of AD use (month +1), this rate further increased to 552.8/10 000 pyrs. After month +1, a trend to lower rates during episodes of AD use was observed among the young as well, albeit inconsistently. The highest rates of suicide as the cause of death were found in month +1 among those aged 40–60 years (53.4/10 000 pyrs) and those aged ≧60 years (42.9/10 000 pyrs), compared to a lower suicide rate among those aged <25 year at month +1 (22.1/10 000 pyrs). Because of the small number of suicide events—sometimes a single case per age and month stratum—no further analyses on suicide were performed. Because of privacy issues and risk of disclosure, no details of these cases are given in Table 3.

### Multivariable Poisson

In a multivariable Poisson regression model, the rates during episodes of AD use per month following the first prescription were compared to the rate during the month prior to the first prescription (month -1, the reference month). This was done for each age category separately by inclusion of terms for age x month interactions. For those aged <25 years, the IRR of suicide attempt during the first month of AD use compared to month -1 was 1.47 (*p* = 0.212) and decreased thereafter. In this age category, the differences by month when testing month as one independent variable were statistically non-significant (overall *p*-value = 0.3055). At higher ages, on the contrary, statistically significant declines in the suicide attempt rate after the start were found, already starting in the first month following the first prescribed AD (e.g. at age 40–60 the IRR at the first month = 0.44, overall *p*-value = 0.0003). The terms for age x month interactions did not reach the level of statistical significance when including age as a categorized variable (*p* = 0.1088, df = 12). When including age as continuous variable, the terms for age x month interactions were highly significant (*p* = 0.0005, df = 4), suggesting an important trend to lower rates of suicide attempts following the start of AD treatment at higher ages, but not, or less so, at younger ages. Similar results were found when excluding those aged <12 years (Table 4).

**Table 4.** Multivariable Poisson Regression Analysis on Suicide Attempt Rates

	Incidence Rate Ratio	95% Confidence Interval	*p*-value
Age <25 years			
Month			
-1	1.00		
+1	1.47	0.80–2.69	0.212
+2	0.70	0.30–1.61	0.407
+3	1.12	0.51–2.43	0.773
+4–6	0.86	0.44–1.67	0.661
Overall effect of month			0.3055 (df = 4)
Age 25–40 years			
Month			
-1	1.00		
+1	0.77	0.46–1.26	0.306
+2	0.41	0.20–0.84	0.015
+3	0.36	0.16–0.83	0.016
+4–6	0.58	0.35–0.96	0.034
Overall effect of month			0.0239 (df = 4)
Age 40–60 years			
Month			
-1	1.00		
+1	0.44	0.24–0.80	0.008
+2	0.35	0.16–0.75	0.008
+3	0.49	0.24–1.03	0.063
+4–6	0.26	0.14–0.51	0.001
Overall effect of month			0.0003 (df = 4)
Age >60 years			
Month			
-1	1.00		
+1	0.56	0.25–1.27	0.170
+2	0.19	0.04–0.86	0.031
+3	0.26	0.06–1.15	0.077
+4–6	0.05	0.007–0.41	0.005
Overall effect of month			0.0082 (df = 4)
Ix {month x age-categorized}			0.1088 (df = 12)
Ix {month x age-continous}			0.0005 (df = 4)

Comparing the months after the start with antidepressants (during episodes of antidepressant use) with the month before the start with antidepressants, by age category.

When restricting the analysis to DBC codes associated with psychiatric consultations in hospitals (codes 0329/01, see Methods) as indicators for suicide attempts, no increase in the rate of suicide attempts during the first month among those aged <25 years was found (IRR = 0.73 [0.28–1.94], *p* = 0.540; differences by month, *p* = 0.2202). Both among those aged <25 years and those age >25 years, a decrease of the suicide attempt (with this restricted definition) rate following the start was found, which was more pronounced and statistically significant among those aged >25 years (for the first month, IRR = 0.37 [0.18–0.78], *p* = 0.009; differences by month, *p* = 0.0004).

When restricting the analysis to episodes of SSRI use among those who started with a SSRI, a small non-significant increase of the suicide attempt rate during the first month among those aged <25 years was found again (IRR = 1.28 [0.63–2.59], *p* = 0.493; differences by month, *p* = 0.5123). At higher ages, a clear decrease immediately after the start with SSRI was observed (e.g. among those aged >40 years, for the first month IRR = 0.36 [0.17–0.75], *p* = 0.006; differences by month, *p* < 0.0001).

When estimating different sets of parameters by gender, an increased suicide attempt rate during the first month was found among females aged <25 years (IRR = 1.94 [0.96–3.92], *p* = 0.065) and a decline in this rate was seen thereafter. For males aged <25 year, no such increase following the start was found (IRR = 0.52 [0.13–2.11], *p* = 0.368). At higher ages, a more pronounced and statistically significant decline in the suicide rate during the months following the start was found for both males and females.

### The First Month After the Start: Comparing Suicide Attempt Cases with Controls

In the first univariable step of the logistic regression on early suicide attempts, age appeared to be a strong predictor, as expected (odds ratio [OR] = 5.18, *p* < 0.001). In the next bivariable steps, a potential predictor was added to the model with age, at first as usual cofactor, and then also with a term for the interaction of predictor x age as effect modifier. Gender as cofactor was not associated with suicide attempts. However, a strong association between age and risk of suicide attempts was found mainly among females (OR = 7.85, *p* < 0.001), whereas the association among males was much weaker and non-significant (OR = 1.61, *p* = 0.433). Thus, gender appeared to be a strong effect modifier. Ethnic origin was not statistically significantly associated with suicide attempt risk, neither as cofactor nor as effect modifier. SSRI as the first prescribed AD was significantly associated with a higher suicide attempt risk (OR = 1.68, *p* = 0.030), but no effect modification could be established (*p* = 0.4612). The effect of age became slightly less after adjustment for use of SSRIs, but was still highly significant (OR = 4.74, *p* < 0.001). A medical specialist as prescriber (vs. a GP) at the start was borderline significantly associated with a higher risk of suicide attempt (OR = 1.53, *p* = 0.072), but this factor did not modify the association with age (*p* = 0.7651). A higher PDD was significantly associated with a higher risk of suicide attempts (*p* = 0.022), but PDD was not an effect modifier. The effect of age became slightly less after adjustment for PDD, but was still highly significant (OR = 4.81, *p* < 0.001). No specific type of AD prescribed at the start (fluoxetine, citalopram, paroxetine, sertraline, escitalopram, mirtazapine, or venlafaxine) was associated with risk of suicide attempts during the first month of treatment, neither as cofactor nor as effect modifier of the association with age.

In Table 5, the final multivariable model is shown. Use of SSRIs was not significantly associated with risk of suicide attempts after adjustment for the other factors, and thus was excluded. After adjustment for prescriber and dosage, the association with age became somewhat less pronounced, but was still strong and statistically highly significant (for females, OR = 6.86, *p* < 0.001; without distinction by gender, OR = 4.52, *p* < 0.001). A strong and statistically significant interaction of age x gender was also found when including all cases with a suicide attempt during months 1–6 following the start and restricting the analysis to those without a registered suicide attempt during the 6 months preceding the start of treatment (for females, OR = 5.41, *p* < 0.001; for males, OR = 2.22, *p* = 0.016; interaction, *p* = 0.023).

**Table 5.** Multivariable Logistic Regression Model to Predict a Suicide Attempt During the Month Following the Start of Antidepressants

	Odds Ratio	95% Confidence Interval	*p*-value
Effect of age (<24 years vs. ≥25 years) by Gender:			
Males	1.43	0.42–4.77	*p* = 0.559
Females	6.86	3.83–12.27	*p* < 0.001
Interaction {Gender x age} (df = 1)			*p* = 0.0212
Gender: Male vs. female	1.28	0.73–2.23	*p* = 0.383
Prescriber: Psychiatrist/other vs. General Practitioner	1.59	0.99–2.53	*p* = 0.051
Prescribed Daily Dose: <0.38	1.00		
0.38–1.00	2.45	1.22–4.92	*p* = 0.011
≥1.00	2.43	1.26–4.68	*p* < 0.001
			*p* = 0.0189 (df = 2)

## Discussion

In this large cohort study with data over a broad age range, we investigated the age dependency of the comparison between the rates of suicide attempts after and the rates of suicide attempts before the start of treatment with ADs. In all age categories, increasing rates during the preceding months until treatment initiation were found. Among those aged >25 years, a consistent decline with statistically significant lower rates immediately after treatment initiation was found. The highest attempt rate during the month prior to initiation was found among those aged <25 years. This rate remained high during the first month following treatment initiation, and was not significantly different from the high rate prior to initiation. After the first month, a trend to lower rates during episodes of actual AD use was observed among the young. The age dependency of attempt rates did not translate into a similar age dependency for rates of suicide as the death cause. The highest rates of suicide were not found among the young, but during the first month of AD treatment among those aged 40–60 and >60 years. The high incidence of suicide behavior during the first month at young ages could not be attributed to characteristics of AD treatment, such as specific class or type of AD or prescribed dosage. However, a strong interaction effect between age and female gender was found that indicated that the high risk at young ages during the early phases of treatment was restricted to the female gender.

Our findings indicate that young AD users are of particular concern for prevention of suicide behavior. Before inferring a causal relationship between use of ADS and suicide behavior at young age, however, a number of critical remarks are needed.

### Comparison with Other Studies

The results of our observational study mirror a recent re-analysis of published and unpublished randomized placebo-controlled trials of fluoxetine and venlafaxine among youths and adults (Gibbons et al., 2012). In the included studies, longitudinally collected rating scales on suicide behavior were used. A favorable treatment response with a reduction of depressive symptoms was found among the young. However, this treatment response was not associated with a concomitant reduction of suicide behavior risk, contrary to expectations. Among adults and geriatric patients, on the other hand, a clear reduction of suicide behavior risk (thoughts of death, ideation, and attempts or completion) was found among those treated with the active substance during the weeks following randomization, which was statistically significantly different from the smaller reduction found among those treated with a placebo. The beneficial effect of ADs on suicide risk was already present after 2 weeks of treatment, in accordance with the observed decline of the suicide attempt rate immediately in the first month after initiation among adults in our analysis. In the meta-analysis, no increase in suicide risk behavior was found among the young during the early phases of treatment. We found a temporally increased rate of attempts early after the start. This increase, however, was not statistically significant (*p* > 0.20). Furthermore, when restricting the analysis to the DBC codes explicitly indicating a suicide attempt (0329/01), our results were in accordance with the findings of Gibbons et al. (2012). In addition, in the logistic regression analysis on early suicide attempt cases, no treatment characteristic, such as type of AD or dosage, could be identified as the culprit of the unfavorable association with young age. Thus, the meta-analysis of Gibbons and our findings suggest that ADs do probably not pave the way for suicidal behavior, but that ADs may be less effective in preventing it at young age.

In a recent well-designed propensity-score matched study using health care utilization data among 162 625 US residents with depression, a high prescribed dose of SSRI was significantly associated with a higher rate of deliberate self-harm (2.2) among those aged <25 years, but equal rates among those aged ≧25 (Miller et al., 2014). This age x dosage interaction effect suggests that those aged <25 years are more sensitive to the unfavorable side-effects of a high AD dose, which may also explain the age dependency of suicide-related behavior during AD treatment. In our analysis, dosage did not appear to explain the high risk of suicide behavior during the early phases of AD treatment among the young. A small part of the age effect disappeared when adjusting for dosage, but no interaction of age x dosage on suicide attempt risk in the multivariable logistic model could be established. Although the finding of Miller et al. (2014) may be regarded as a strong argument in favor of the presence of an adverse causal effect of ADs specifically associated with young age, confounding by indication may still explain a part of the results. Furthermore, the relevance of deliberate self-harm for risk of suicide attempts and death remains a question (Barbui and Patten, 2014).

Observational investigators may eschew before-and-after comparisons, especially to avoid the pitfall that suicidal behavior leads physicians to start with AD treatment, and often focus on differences between various classes of AD (Jick et al., 2004; Schneeweiss et al., 2010). By including a very short time interval prior to the first prescription, the observation of a spuriously reverse association between use of ADs and suicide behavior may be avoided. Indeed, we found high rates of suicide attempts during the month immediately preceding the first recorded AD, probably indicative of the worsening of the clinical condition that gave rise to the decision to start with ADs. The rates of suicide attempt were still high during the first month of AD treatment in all age categories, but decreased further thereafter. This decrease was more pronounced and statistically significant among those aged >25 years. This finding is in accordance with the case-control study by Jick and colleagues. In that study, a strong association between a shorter duration since the first recorded AD and a higher risk of suicide behavior was found, and this association was increasingly prominent with increasing age (*p* = 0.025 for trend; Jick et al., 2004). In a study of the Veteran Health Administration among 226 866 veterans with an incident depressive disorder in 2003 and 2004, a decrease in the risk of suicide attempts after the first recorded AD in comparison with the period before the first prescription was found (Gibbons et al., 2007b). A similar decrease was found among those aged 18–25 years, without an indication for an interaction of age x episode. An important limitation of this study was that the observation periods before and after treatment initiation incorporated long time intervals (>200 days) without sub-division into months. In addition, for the observation period following treatment initiation, no subdivision was made in episodes of actual use or episodes of no use. In the study of Simon et al. (2006), a more refined before-and-after comparison was employed by a subdivision of the observation time into 1-month episodes. The highest rates of suicide attempt were found during the month prior to initiation, with a subsequent decline in these rates during the six 1-month intervals thereafter both among the young (<18 years) and the older patients (≧18 years). This pattern was also found in a subsequent analysis among patients treated with psychotherapy, and thus is probably not specific for treatment with ADs (Simon and Savarino, 2007). This pattern was not mirrored in a similar decrease of suicidal events. This indicates that attempt risk does not necessarily reflect suicide death risk, as was also found in the present study. In the study by Simon et al. (2006), however, no subdivision in episodes of actual use and intermittent episodes of no use after treatment initiation was made. Therefore, subtle differences in time patterns between age categories during actual use may have been obscured.

### Strengths and Limitations

Following the start of AD use, patients often stop usage temporarily or permanently, causing the comparison of episodes of actual use with episodes of no use in our study is relevant for treatment trajectories. In accordance with the abovementioned observational studies of Gibbons et al. (2007b) and Simon et al. (2006), we found a clear decrease in attempt rates during the months following initiation. This was observed in all age categories (Figure 1–4). However, when assessing the rates of suicide attempts during episodes of actual use (Figure 1–4, dark bars), differences between age categories became visible, with a high persistent attempt rate during the first month of treatment and a less prominent decrease thereafter among the young (<25 years). Thus, our study may be regarded as a further refinement of earlier analyses, making it possible to track and analyze suicide attempt cases that were possibly causally related to adverse effects of AD use during the very early stages after the start with ADs.

The observed age dependency for suicide attempts may have been confounded by indication. That is, younger patients being treated with AD may have a more severe disorder at the time of prescription, other diagnoses with higher suicide attempt rates, and/or a higher risk of treatment resistance compared to older patients being treated with ADs. Indeed, the highest suicide attempt rates prior to treatment initiation and a higher percentage of patients with a record in specialist treatment were found among the young. A major limitation of the study is that specific diagnoses and disease severity at the time of treatment initiation were not taken into account. An important part of the ADs was prescribed by GPs who do not register the diagnosis in the AHD, and DBC codes do not contain clinical severity scales. However, in the logistic regression model focusing on early suicide attempt cases, the strong age dependency was still present after taking into account dosage, specific type of AD, and prescriber (specialist vs. GP), probably all important indicators of disease severity. Furthermore, even if the found age dependency for suicide attempts and the persistence of the high risk during the early treatment phases at young ages are (partially) explained by confounding by indication, the finding of no similar age dependency for suicide as cause of death is more remarkable and stronger.

On one hand, a number of suicide attempts have probably been missed, as only a part of suicide attempts (about a third) get medical attention and are treated (Eaton et al., 2012). On the other hand, a number of defined suicide attempts in our study may have been misclassified, especially those that were related to a DBC code for diagnosis of (auto-)intoxication. Still, poisoning by toxic substances is the most common method of suicide attempt leading to hospital admission and may be regarded as a valid indicator of suicide attempts at the population level (Patrick et al., 2010; Lu et al., 2014). Although the non-significant initial increase of attempt rates among the young could not be reproduced, a similar age dependency was found when restricting the definition of attempt to the DBC codes for psychiatric consultation because of a suicide attempt (0329/01). Although establishment of suicide attempts using administrative data is prone to underestimation and misclassification, this probably did not influence the observed age dependency in estimated relative risks.

Both in the Poisson regression model of attempt rates and the logistic regression on correlates of early suicide attempts, gender appeared to be an important effect modifier of the age dependency. A remarkable finding was that no differences in suicide attempt rates between males and females at age <25 years were found during the month prior to the start. Further analyses on other databases are required to find out whether the effect modification of gender is a reproducible finding and reflects a differential sensitivity for the adverse and/or beneficial effects of ADs or is a behavioral phenomenon associated with the underlying disorder. When clinically monitoring young users of AD for risk of suicidal behavior, the clinician should be alert to the high risk among female patients during the early phases of treatment.

In conclusion, we found high rates of suicide attempts among young users of ADs, both before and after treatment initiation, which clearly contrasted with the lower rates and clear decreases in these rates after treatment initiation among those aged >25 years. While the presence of a causal effect of ADs on the risk of suicide attempts in some vulnerable patients during the early phases of AD treatment cannot be precluded definitely, our results do not univocally show an increase following the start, but are in accordance with the notion that ADs seem less effective in preventing suicidal behavior at young age. As no similar age dependency for suicidal events was found, and even the highest risks of suicide were found during the early treatment phases among those aged >40 years, episodes of AD use among the young do not necessarily predict a high risk of suicide death.

## Statement of Interest

None.
